# Plant Metabolic Engineering Strategies for the Production of Pharmaceutical Terpenoids

**DOI:** 10.3389/fpls.2016.01647

**Published:** 2016-11-08

**Authors:** Xu Lu, Kexuan Tang, Ping Li

**Affiliations:** ^1^State Key Laboratory of Natural Medicines, China Pharmaceutical UniversityNanjing, China; ^2^Plant Biotechnology Research Center, School of Agriculture and Biology, Shanghai Jiao Tong UniversityShanghai, China

**Keywords:** medicinal plants, metabolic regulation, elicitors, overexpression, suppression, transcription factors, endogenous phytohormones, ectopic expression

## Abstract

Pharmaceutical terpenoids belong to the most diverse class of natural products. They have significant curative effects on a variety of diseases, such as cancer, cardiovascular diseases, malaria and Alzheimer’s disease. Nowadays, elicitors, including biotic and abiotic elicitors, are often used to activate the pathway of secondary metabolism and enhance the production of target terpenoids. Based on *Agrobacterium*-mediated genetic transformation, several plant metabolic engineering strategies hold great promise to regulate the biosynthesis of pharmaceutical terpenoids. Overexpressing terpenoids biosynthesis pathway genes in homologous and ectopic plants is an effective strategy to enhance the yield of pharmaceutical terpenoids. Another strategy is to suppress the expression of competitive metabolic pathways. In addition, global regulation which includes regulating the relative transcription factors, endogenous phytohormones and primary metabolism could also markedly increase their yield. All these strategies offer great opportunities to enhance the supply of scarce terpenoids drugs, reduce the price of expensive drugs and improve people’s standards of living.

## Introduction

Terpenoids are the most diverse class of natural products and over 40,000 different terpenoids have been described (Misawa, 2011). Many of them are isolated from medicinal plants, such as *Ginkgo biloba, Taxus chinensis, Artemisia annua, Salvia miltiorrhiza*, and *Panax ginseng.* Terpenoids from plants, such as artemisinin, taxol and ginkgolides, have good curative effects on a variety of diseases. Artemisinin, a sesquiterpenoids lactone derived from *A. annua*, is currently the best therapeutic against both drug-resistant and cerebral malaria-causing strains of *Plasmodium falciparum* (Weathers et al., 2006). Paclitaxel (taxol), a diterpenoid produced by *Taxus brevifolia* and other *Taxus*-species is an important anticancer agent (Lenka et al., 2012). Ginkgolides, a structurally unique family of diterpenoids, are highly specific platelet-activating factor receptor antagonists (Stromgaard and Nakanishi, 2004).

However, at present the commercialization of pharmaceutical terpenoids is limited due to their low yield from natural sources (Misawa, 2011). Many medicinal plants grow slowly and are susceptible to environmental conditions. Some medicinal plants are endangered and cannot be harvested for isolation of pharmaceutical terpenoids. Furthermore, the contents of pharmaceutical terpenoids usually show large fluctuations, which is not conducive to the extraction and quality control of drugs. Great efforts have been made to enhance the production of pharmaceutical terpenoids. However, the total chemical synthesis of these complicated compounds is costly. While most of pharmaceutical terpenoids biosynthetic pathways have not been resolved, the biosynthesis in microorganisms is very difficult. Consequently, researchers have carried out a number of studies on plant metabolic engineering of pharmaceutical terpenoids (**Table 1**). This review covers the plant metabolic engineering strategies for the production of pharmaceutical terpenoids from 2009 to July 2016.

**Table 1:** Pharmaceutical terpenoids production by plant metabolic engineering strategies.

Classes	Metabolites	Medicinal plants	Strategy
Monoterpenoids	Linalool	*L. latifolia*	Overexpression (Mendoza-Poudereux et al., 2014)
	Geraniol	*N. tabacum*	Overexpression (Ritala et al., 2014); Global regulation (Vasilev et al., 2014)
	Loganic acid; Secologanin	*C. roseus*	Global regulation (Van Moerkercke et al., 2015, 2016)
Sesquiterpenoids	Artemisinin	*A. annua*	Overexpression (Lu et al., 2013a); Suppression (Zhang et al., 2009; Lv et al., 2016); Global regulation (Yu et al., 2012; Lu et al., 2013b, 2014; Zhang et al., 2013, 2015; Han J.L. et al., 2014; Ji et al., 2014; Tan et al., 2015; Shen et al., 2016)
	Dihydroartemisinic acid	*A. annua*	Global regulation (Lu et al., 2013b, 2014; Shen et al., 2016); Suppression (Lv et al., 2016)
	Artemisinic acid	*A. annua*	Global regulation (Yu et al., 2012; Lu et al., 2014; Shen et al., 2016)
	Valencene	*N. benthamiana*	Suppression (Cankar et al., 2015)
	Bilobalide	*G. biloba*	Elicitors (Kang et al., 2009)
	Farnesene; Bergamotene	*N. attenuata*	Overexpression (Schuman et al., 2014)
Diterpenoids	Paclitaxel	*T. chinensis*; *T. media*	Elicitors (Li et al., 2009; Xu et al., 2011; Zhou and Zhong, 2011; Onrubia et al., 2013)
	Taxanes	*T. cuspidata*; *C. avellana*; *T. chinensis*; *T. media*	Elicitors (Patil et al., 2014; Gallego et al., 2015); Global regulation (Dong et al., 2015); Combined (Expósito et al., 2010)
	Taxadiene	*N. benthamiana*	Overexpression (Hasan et al., 2014)
	Tanshinones	*S. miltiorrhiza* *S. castanea*	Overexpression (Kai et al., 2011); Global regulation (Gu et al., 2012)
	Ginkgolides	*G. biloba*	Elicitors (Kang et al., 2009)
	Andrographolide	*A. paniculata*	Elicitors (Praveen et al., 2009; Gandi et al., 2012)
Triterpenoids	Ginsenosides	*P. ginseng*	Elicitors (Huang and Zhong, 2013); Overexpression (Shim et al., 2010; Han et al., 2013; Kim et al., 2014)
	Maytenin	*P. campestris*	Elicitors (Paz et al., 2013)
	Platycoside	*P. gradiflorum*	Overexpression (Kim Y.K. et al., 2013)
	Dammarenediol-II; Protopanaxadiol	*N. tabacum*	Overexpression (Han J.Y. et al., 2014; Chun et al., 2015)
Tetraterpenoids	Carotenoids	*I. batatas*; *S. lycopersicum*	Suppression (Kim S.H. et al., 2013); Global regulation (Sun et al., 2012)
	Lycopene	*S. lycopersicum*	Global regulation (Sun et al., 2012)

## The Biosynthetic Pathways of Pharmaceutical Terpenoids in Medicinal Plants

**FIGURE 1:**
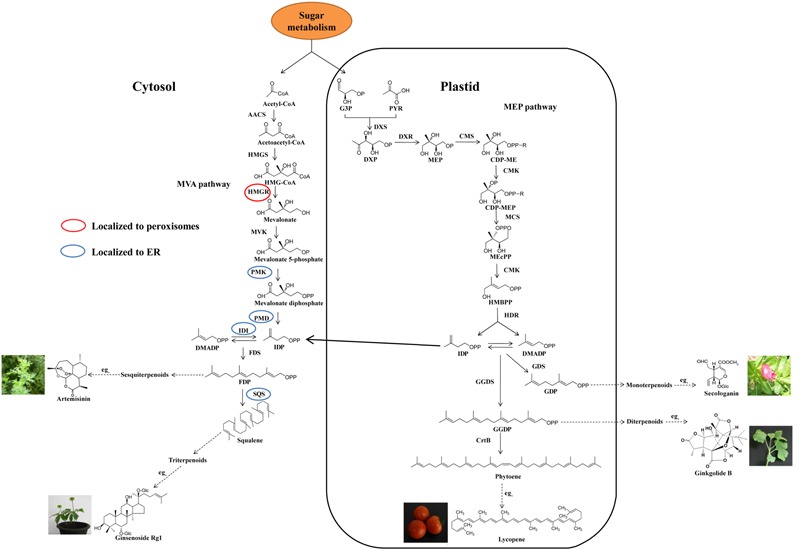
**Biosynthetic pathway of pharmaceutical terpenoids.** MVA, mevalonic acid; MEP, methylerythritol phosphate; PYR, pyruvate; G3P, glyceraldehyde-3-phosphate; IDP, isopentenyl diphosphate; DMADP, dimethylallyl diphosphate; FDP, farnesyl diphosphate; GDP, geranyl diphosphate; GGDP, geranylgeranyl diphosphate; ER, endoplasmic reticulum; DXP, 1-deoxy-D-xylulose-5-phosphate; DXS, DXP synthase; MEP, 2-*C*-methyl-D-erythritol-4-phosphate; DXR, DXP reductase; CDP-ME, 4-diphosphocytidyl-2-*C*-methyl-D-erythritol; CMS, CDP-ME synthase; CDP-MEP, CDP-ME 2-phosphate; CMK, CDP-ME kinase; MEcPP, 2-*C*-methyl-D-erythritol-2,4-cyclodiphosphate; MCS, MEcPP synthase; HMBPP, 1-hydroxy-2-methyl-2-(E)-butenyl-4-diphosphate; HDS, HMBPP synthase; HDR, HMBPP reductase; AACS, acetoacetyl-CoA synthase; HMGS, hydroxymethylglutaryl (HMG)-CoA synthase; HMGR, HMG-CoA reductase; MVK, mevalonate kinase; PMK, phosphomevalonate kinase; PMD, mevalonate diphosphate decarboxylase; IDI, isopentenyl diphosphate isomerase; GDS, geranyl diphosphate synthase; FDS, farnesyl diphosphate synthase; GGDS, geranylgeranyl diphosphate synthase; SQS, squalene synthase; CrtB, phytoene synthase.

Despite the complex structure, terpenoids are biosynthesized from the basic isoprene units, i.e., isopentenyl diphosphate (IDP) and dimethylallyl diphosphate (DMADP). In plants, there are two independent pathways to produce IDP and DMADP (**Figure 1**). One is the methylerythritol phosphate (MEP) pathway, which is localized in the plastids and starts with the biosynthesis of 1-deoxy-D-xylulose 5-phosphate (DXP) from D-glyceraldehyde 3-phosphate (GAP) and pyruvate (PYR) (Banerjee and Sharkey, 2014). The MEP pathway, which comprises seven enzymatic steps, is mainly regulated by 1-deoxy-D-xylulose-5-phosphate synthase (DXS) and 1-deoxy-D-xylulose-5-phosphate reductoisomerase (DXR) (Weathers et al., 2011). The other pathway is the classical mevalonic acid (MVA) pathway comprised of six enzymatic steps, which are localized to the cytosol, endoplasmic reticulum (ER) and peroxisomes (Gutensohn et al., 2014). 3-Hydroxy-3-methylglutaryl-CoA reductase (HMGR) localized to peroxisomes is considered as a rate-limiting step (Opitz et al., 2014) (**Figure 1**). There is an unidirectional proton symport system between the cytosol and the plastid resulting in transport of C_5_-precursors between the two compartments (Bick and Lange, 2003).

The intermediates IDP and DMADP are condensed to a series of linear terpenoid intermediates by prenyl transferases, which are the precursors of different terpenoids. The terpenoids are sorted into classes depending on the number of isoprene units: monoterpenoids (C_10_), sesquiterpenoids (C_15_), diterpenoids (C_20_), triterpenoids (C_30_), and tetraterpenoids (C_40_), as shown in **Figure 1** (Smanski et al., 2012; Sato, 2013). IDP and DMADP are condensed to the C_10_-compound geranyl diphosphate (GDP) by geranyl diphosphate synthase (GDS) in the plastid. In the cytosol, farnesyl diphosphate synthase (FDS) converts two molecules of IDP and one molecule of DMADP to the C_15_-compound farnesyl diphosphate (FDP), which is the precursor of sesquiterpenoids. Two molecules of FDP can be condensed to squalene (the precursor of triterpenoids) by squalene synthase (SQS) localized to the ER membrane through a short C-terminal membrane-spanning sequence. For the biosynthesis of diterpenoids in the plastid, geranylgeranyl diphosphate synthase (GGDS) can build the precursor of diterpenoids, geranylgeranyl diphosphate (GGDP), by condensing three molecules of IDP and one molecule of DMADP. Finally, two molecules of GGDP can form the precursor of phytoene (tetraterpenoid), in the plastid (Loto et al., 2012).

Subsequently, different terpene synthases (TPSs) use the linear prenyl compounds as substrate to start the biosynthesis of specific terpenoids. In the biosynthesis of pharmaceutical terpenoids, further modifications are catalyzed by various cytochrome P450-dependent oxidoreductases (CYP450s), acyltransferases, glucosyltransferases and dehydrogenases. Recently, more and more key genes of pharmaceutical terpenoid biosynthetic pathways have been cloned from different medicinal plants (Guo et al., 2013; Miettinen et al., 2014; Yan et al., 2014; Moses et al., 2015). Artemisinin, a sesquiterpenoids lactone, is a natural antimalarial drug and its discovery was awarded the 2015 Nobel Prize in Physiology or Medicine. After extensive investigations during the last years, the artemisinin biosynthetic pathway is almost completely resolved (Bouwmeester et al., 1999; Brown and Sy, 2004; Teoh et al., 2006, 2009; Zhang et al., 2008). All studies mentioned above provide the theoretical support to plant metabolic engineering studies for the production of pharmaceutical terpenoids.

## Plant Metabolic Engineering Strategies for Production of Pharmaceutical Terpenoids

### Elicitors

**FIGURE 2:**
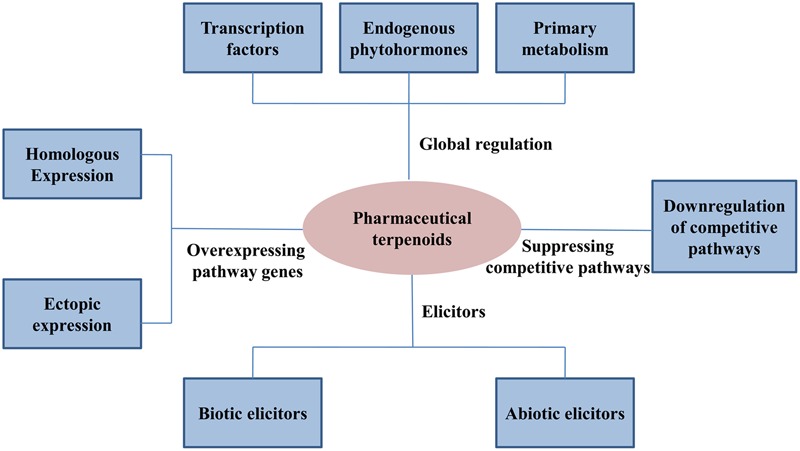
**Plant metabolic engineering strategies to regulate pharmaceutical terpenoids**.

Pharmaceutical terpenoids of medicinal plants are often natural defense metabolites against pathogen attacks. Elicitors, including biotic and abiotic elicitors, can be used to activate the pathway of secondary metabolism and enhance the production of target terpenoids (**Figure 2**).

#### Biotic Elicitors

Biotic elicitors are obtained from living organisms such as bacteria, viruses and fungi. Plant hormones (salicylic acid, jasmonates, abscisic acid etc.) are involved in the response to elicitors and can be used as substitutes for biotic elicitors (Cusido et al., 2014). Several biotic elicitors have significant functions in promoting the production of pharmaceutical terpenoids. Kang et al. (2009) reported that a native bacterial elicitor effectively increased the secondary metabolite accumulations in *G. biloba* cell suspension cultures. After 24 h treatment with native *Staphylococcus aureus*, the bilobalide, ginkgolide A and ginkgolide B levels were enhanced 2.6-, 1.5- and 2.1-fold, respectively, compared to the untreated control. Praveen et al. (2009) established an adventitious root culture system from leaf segments of *Andrographis paniculata* on Murashige and Skoog (MS) medium with α-naphthaleneacetic acid (NAA). The content of andrographolide (diterpenoid) was 3.5-fold higher compared to the natural cells in suspension culture. Adventitious root cultures of *Peritassa campestris* (Celastraceae) were established from seed cotyledons cultured in semisolid Woody Plant Medium (WPM) supplemented with sucrose, PVP and IBA. The highest content of maytenin (a quinone-methide triterpenoid) in adventitious root culture was 972 μg/g DW after cultivation for 7 days (Paz et al., 2013). It was 5.55-fold higher than that found in the roots of seedlings grown in a greenhouse. Biotic elicitors are also important tools to produce the important anti-cancer drug paclitaxel. A 20-L bioreactor was used for paclitaxel production by co-culturing *Taxus chinensis* suspension cells and its endophytic fungi, *Fusarium mairei*. Within co-culture of 15 days, 25.6 mg/L of paclitaxel was produced. The productivity is 1.71 mg/L per day and 38-fold higher than that by uncoupled culture (0.68 mg/L within 15 days) (Li et al., 2009). During the cell culture process, different biotic elicitors are used to enhance the production of paclitaxel. Several labs focused their studies on selecting a more powerful elicitor for inducing paclitaxel biosynthesis in suspension cell lines. The results showed that coronatine, methyl jasmonate (MeJA), salicylic acid (SA) and abscisic acid (ABA) are useful elicitors that can affect the biosynthesis of paclitaxel and related taxanes (Li et al., 2009; Xu et al., 2011; Zhou and Zhong, 2011; Onrubia et al., 2013). Recently, some researchers reported that coronatine and MeJA treatments may repress the growth of suspension cells. One example is cell suspension cultures of *Corylus avellana* producing taxanes. Treatments with MeJA or coronatine resulted in a statistically significant reduction in suspension cells growth from 11.5 g DCW/L to 4.28 g DCW/L and 5.69 g DCW/L, respectively, after 14 days, while the total taxane content was increased 3- and 27-fold, respectively (Gallego et al., 2015). In another study on the effects of MeJA on cell suspension culture of *Taxus cuspidata*, the growth of MeJA treated cultures decreased from 14 g DW/L to 8 g DW/L (Patil et al., 2014). Essentially no taxanes were produced in the untreated cell cultures but after MeJA-treatment the yield of paclitaxel was 2.5 mg/g DW of cells. Consequently, despite affecting the growth of suspension cells, MeJA or coronatine may be effectively used for increased paclitaxel production in *Taxus* cell suspension cultures.

#### Abiotic Elicitors

Abiotic elicitors include inorganic compounds (copper sulfate, silver nitrate, etc.) and metal ions (Cusido et al., 2014). There are only a few reports on the use of abiotic elicitors for the production of pharmaceutical terpenoids in cell suspension cultures. Andrographolide, a diterpenoid lactones, exhibits important pharmacological activities such as anticancer, anti-HIV, and anti-inflammatory (Srivastava and Akhila, 2010). Gandi et al. (2012) reported that abiotic elicitors (CdCl_2_, AgNO_3_, CuCl_2_ and HgCl_2_) were used to enhance the andrographolide content in suspension cultures of *A. paniculata.* Among all those metal salts, the treatment with CdCl_2_ had the strongest effect on andrographolide production, which could reach 6.3 mg/g DCW, whereas the control accumulated 1.5 mg/g DCW.

The use of abiotic elicitors was an effective strategy for increasing the production of triterpenoid ginsenosides in suspension cultures of *P. ginseng*. Huang and Zhong (2013) described that heavy metal salts, including NaVO_3_, NH_4_VO_3_, NiSO_4_, VOSO_4_, CuSO_4_ and MnSO_4_, were used to induce ginsenoside biosynthesis. Vanadate was demonstrated to be the most efficient of all the treatments tested. After 4 days treatment with vanadate, the highest ginsenoside (Rg_1_ + Re + Rb_1_ + Rc + Rd) content was 5.6 mg/g DW (day 14). Further research showed that vanadate treatment induced the endogenous JA biosynthesis and up-regulated the transcription levels of *SQS*, squalene epoxidase (*SE*) and dammarenediol-II synthase (*DS*) genes.

### Homologous Overexpression of Pharmaceutical Terpenoid Biosynthesis Key Genes

#### Overexpression of Single Gene

As pharmaceutical terpenoid production is strictly controlled by enzymes of the biosynthetic pathways, one way to increase the productivity is to regulate the expression of such genes (De Geyter et al., 2012). Ginsenosides, a group of triterpenoids, which can be divided into protopanaxadiols (Rb_1_, Rb_2_, Rc and Rd) and protopanaxatriols (Re, Rf, and Rg_1_), are the main pharmacological active constituents of *P. ginseng* (Qi et al., 2011). During recent years, several genes of the ginsenosides biosynthetic pathway have been cloned and overexpessed in *P. ginseng*. Overexpression of ginsenosides biosynthetic pathway key gene *PgSQS1*, which can up-regulate the expression of SE, β-amyrin synthase (β-AS) and cycloartenol synthase (CAS), resulted in a twofold increase of phytosterols and 1.6- to 3-fold increase of total ginsenosides in transgenic ginseng adventitious root cultures. However, the growth rate of transgenic ginseng roots is slower than that of non-transgenic adventitious roots (Shim et al., 2010). Han et al. (2013) described that overexpression of ginsenoside biosynthesis key gene *CYP716A52v2* greatly enhanced the content of oleanane-type ginsenoside (ginsenoside Ro) in *P. ginseng* plants, while the levels of other dammarene-type ginsenosides were similar to the control lines. By *A. tumefaciens*-mediated transformation, the *PgHMGR* gene was overexpressed and enhanced the accumulation of ginsenosides 1.5- to 2-fold in transgenic ginseng adventitious root cultures (Kim et al., 2014).

#### Co-expression of Multiple Genes

Metabolic regulation of multiple key genes in biosynthetic pathways can effectively increase the pharmaceutical terpenoids content in medicinal plants. Tanshinones are abietane-type norditerpenoid quinines in *S. miltiorrhiza*. They have antibacterial, anti-inflammatory effects and broad antitumor activities (Dong et al., 2011; Gao et al., 2014). The introduction of the *SmHMGR* and/or *SmGGDS gene*, as well as *SmDXS* gene in *S. miltiorrhiza* hairy root lines result in a significant enhancement of tanshinone production (Kai et al., 2011). Co-expression of the *SmHMGR* and *SmGGDS* genes resulted in the highest production of tanshinone (about 2.7 mg/g DW), which was about 4.7-fold higher than the control (0.475 mg/g DW). Overexpression of artemisinin biosynthesis genes *ADS* (Amorpha-4,11-diene synthase gene), *CYP71AV1* (cytochrome P450-dependent hydroxylase gene) and *CPR* (NADPH: cytochrome P450 oxidoreductase gene) promoted the accumulation of artemisinin in *A. annua.* The artemisinin content could reach 15.1 mg/g dry weight (DW), which was 2.4-fold higher than the control plants (Lu et al., 2013a).

### Ectopic Expression of Biosynthetic Genes to Produce Pharmaceutical Terpenoids

Some medicinal plants, which produce low yields of pharmaceutical terpenoids in native plants, are hard to genetically transform. Several researchers have used ectopic expression of terpenoid synthases/cyclases (TPSs) to produce pharmaceutical terpenoids in different plants. Geraniol is a monoterpenoid alcohol, with important commercial value in fragrance industries due to its pleasant rose-like odor, and it can also be used as an anticancer drugs and antimicrobial reagents (Unlu et al., 2010; Polo et al., 2011). A geraniol synthase gene from *Valeriana officinalis* (*VoGES*) was engineered to produce geraniol in tobacco hairy root cultures. GC–MS analysis revealed that the free geraniol content in 20 hairy root cultures was with an average of 13.7 μg/g DW and the maximum was 31.3 μg/g DW. Metabolic analysis revealed that there were six major glycoside forms of geraniol derivatives. After deglycosylation, the total geraniol levels were up to 204 μg/g DW (Ritala et al., 2014). High content of linalool and low content of camphor are desired in *Lavandula latifolia* (spike lavender) oils for the perfume and cosmetic industries (Kaloustian et al., 2000). The linalool synthase (*LIS*) gene from *Clarkia breweri*, encoding the linalool synthase was overexpressed in *L. latifolia* plants. The linalool content was increased significantly in the transgenic *L. latifolia* young leaves, where the linalool content increased up to 10-fold (Mendoza-Poudereux et al., 2014). Maize (*Zea mays*) terpene synthase10 (*ZmTPS10*), which produces the sesquiterpenes (E)-β-farnesene and (E)-α-bergamotene, was ectopically overexpressed in *Nicotiana attenuata*. Transgenic *N. attenuata* plants contained 2- to 25-fold more (E)-α-bergamotene than the wild-type plants. Jasmonate elicitors or herbivore treatment could further induce an increased emission of (E)-β-farnesene and (E)-α-bergamotene in the transgenic *N. attenuata* plants (Schuman et al., 2014).

Taxadiene, which is the first committed product of paclitaxel biosynthesis, is produced by taxadiene synthase (TS) from GGDP. Hasan et al. (2014) constitutively overexpressed the *TbTS* gene in *N. benthamiana.* Transformed *N. benthamiana* line produced 11–27 μg/g DW taxadiene. The plants were further treated with MeJA, and the accumulation of taxadiene increased to 35 μg/g DW. Ectopic overexpression of other terpenoid biosynthesis genes may also increase the productivity of triterpenoids. By *Agrobacterium rhizogenes*-mediated ectopic transformation, overexpression of *Panax ginseng HMGR1* (*PgHMGR1*) resulted in 1.1- to 1.6-fold increase of phytosterols and 1.5- to 2.5-fold higher platycoside yields in *Platycodon gradiflorum* hairy root cultures (Kim Y.K. et al., 2013). Several researchers overexpressed ginsenoside biosynthesis key gene *PgDS* or *PgDS* plus *PgCYP716A47* in transgenic tobacco plants. Overexpression of *PgDS* gene resulted in more dammarenediol-II accumulated in transgenic tobacco roots than stems, leaves and flower buds. Dammarenediol-II production could reach 158 μg/g DW in the root of transgenic line. And its accumulation in the cell suspension culture could reach 573 μg/g DW after 3 weeks of culture (Han J.Y. et al., 2014). After co-overexpressing *PgDS* and *PgCYP716A47* (CYP450 gene of Ginsenosides biosynthesis pathway) in tobacco, the concentration of protopanaxadiol (PPD) was 2.3–5.7 μg/g DW in transgenic tobacco leaves. 2,4-D treatment could increase the expression of *HMGR* and *SE* in cell suspension culture. The production of PPD in a 250-ml shake flask culture or in a 5-L airlift bioreactor culture was 167 and 981 μg/g DW, respectively (Chun et al., 2015).

### Suppression of Competitive Pathways to Increase the Production of Pharmaceutical Terpenoids

Another strategy is to suppress the expression of competitive metabolic pathways. The sterol pathway is a competitive pathway of artemisinin biosynthesis in *A. annua*. Downregulation of the expression of *SQS*, a key gene of sterol pathway, by RNA interference (RNAi), resulted in a significant increase of artemisinin content in transgenic plants, with the highest values reaching 31.4 mg/g DW, which is about 3.14-fold higher than the control plants (Zhang et al., 2009). Four competitive branch pathway genes β-caryophyllene synthase gene (*CPS*), β-farnesene synthase gene (*BFS*), germacrene A synthase gene (*GAS*) and *SQS* were further down-regulated independently by the antisense method in *A. annua*. The content of artemisinin and dihydroartemisinic acid (DHAA) were increased significantly in different transgenic lines. In anti-CPS transgenic plants, the contents of artemisinin and DHAA were increased by 77 and 132%, respectively. In anti-BFS transgenic plants, the contents of artemisinin and DHAA were increased by 77 and 54%, respectively. In anti-GAS transgenic plants, the contents of artemisinin and DHAA were enhanced by 103% and 130%, respectively. In anti-SQS transgenic plants, the contents of artemisinin and DHAA were enhanced by 71 and 223%, respectively (Lv et al., 2016). Carotenoids (tetraterpenes), which exert a wide range of functions in the plant kingdom, are required for human health (Mo and Elson, 1999). To increase carotenoid biosynthesis via the β-branch-specific pathway, the expression of lycopene 𝜀-cyclase (LCY-𝜀) gene, which is the first gene of competitive branch pathway, was downregulated by RNAi (Kim S.H. et al., 2013). It was shown that the β-carotene content was approximately 21-fold higher in the sweetpotato transgenic calli than in the control, whereas the lutein content was reduced to levels undetectable in the transgenic calli. The (+)-valencene is an aroma sesquiterpenoid. Silencing the endogenous 5-*epi*-aristolochene synthase gene (*EAS*) and *SQS*, which are competing for the FDP pool, by RNAi resulted in a 2.8-fold increased yield of (+)-valencene in *N. benthamiana* plants (Cankar et al., 2015).

### Regulating the Expression of Transcription Factors

Transcription factors have important functions in controlling the transcription of biosynthetic genes and they may constitute important tools to regulate the production of secondary metabolites in plants (Vom Endt et al., 2002) (**Figure 2**). Several types of transcription factors have already showed to have global regulation functions in pharmaceutical terpenoids.

#### AP2/ERF Transcription Factors

AP2/ERF transcription factors, which carry a conserved binding domain of 57–66 amino acids, are involved in plant response to biotic and abiotic stresses, as well as in the regulation of metabolism in various plant species (Agarwal et al., 2006). Recently, AP2/ERF transcription factors have received attention in artemisinin metabolic engineering. Overexpression of *AaERF1* and *AaERF2*, which are able to bind to the CRTDREHVCBF2 (CBF2) and RAV1AAT (RAA) motifs of *ADS* and *CYP71AV1* promoters, enhanced the contents of artemisinin and artemisinic acid in transgenic *A. annua* plants (Yu et al., 2012). Overexpression of a trichome-specific AP2/ERF transcription factor *AaORA* resulted in a significant increase in artemisinin and DHAA. The disease resistance to *Botrytis cinerea* was also increased in these transgenic *A. annua* plants (Lu et al., 2013b). Another AP2/ERF transcription factor, TRICHOME AND ARTEMISININ REGULATOR 1 (TAR1), which binds to the *cis*-acting elements of *ADS* and *CYP71AV1* promoters, was cloned from *A. annua*. RNAi of *TAR1* caused a decreased accumulation of artemisinin and abnormal phenotype of glandular specific trichomes (GSTs) and T-shaped non-glandular trichomes in transgenic *A. annua* plants, as well as altered cuticular wax load. On the other hand, overexpression of *TAR1* markedly increased the content of artemisinin in transgenic *A. annua* lines (Tan et al., 2015).

#### WRKY Transcription Factors

Transcription factors of WRKY family, which can specifically bind to the W-box (TTGACC/T) of promoters, are involved in regulating defense responses and developmental and physiological processes of plants, such as trichome initiation, senescence and metabolism (Rushton et al., 2010). The *AaWRKY1* gene was cloned from a GST cDNA library of *A*. *annua* (Ma et al., 2009). The trichome-specific overexpression of *AaWRKY1* effectively improved the transcription of *CYP71AV1*, i.e., up to 33 times as compared to the wild-type plants. However, the transcription levels of *FDS, ADS* and *DBR2* (a double bond reductase 2 in artemisinin biosynthesis pathway) did not change significantly in these transgenic *A. annua*. However, the significantly up-regulated *CYP71AV1* increased the production of artemisinin by about 1.8-fold in transgenic plants (Han J.L. et al., 2014).

#### Basic Helix–Loop–Helix (bHLH) Transcription Factors

The basic helix–loop–helix (bHLH) transcription factors are found in all eukaryotic organisms and are involved in a myriad of regulatory processes. Iridoids and (seco)iridoids (including loganic acid and secologanin) are monoterpenoids. Many of these compounds are bioactive themselves, with among others anticancer, antimicrobial and anti-inflammatory activities (Dinda et al., 2007a,b; Viljoen et al., 2012). The jasmonate-regulated bHLH transcription factor (bHLH iridoid synthesis 1, BIS1) was cloned from *Catharanthus roseus*. BIS1 could transactivate the expression of the genes involved in the conversion of the terpenoid precursor GDP to iridoids loganic acid and secologanin. Overexpression of *BIS1* was effective to boost production of high-value iridoids and monoterpenoid indole alkaloids (MIAs) in *C. roseus* suspension cell cultures (Van Moerkercke et al., 2015). A jasmonate (JA)-responsive bHLH TF BIS2 could transactivate promoters of iridoid biosynthesis genes and can homodimerise or form heterodimers with BIS1. Stable overexpression of BIS2 in *C. roseus* suspension cells and transient ectopic expression of BIS2 in *C. roseus* petal limbs resulted in increased transcript accumulation of methylerythritol-4-phosphate and iridoid pathway genes (Van Moerkercke et al., 2016). A bHLH transcription factor from *A. annua*, AabHLH1, was cloned from a GST cDNA library. The AabHLH1 protein binds to the E-box *cis*-elements in both *ADS* and *CYP71AV1* promoters, and possessed transactivation activity in yeast. Transient expression of *AabHLH1* in *A. annua* leaves increased transcript levels of *ADS, CYP71AV1* and *HMGR*, which are all involved in artemisinin biosynthesis (Ji et al., 2014). Recently, *AaMYC2*, which was rapidly induced by JA and could bind to the G-box like motifs in the promoters of *CYP71AV1* and *DBR2*, was cloned. Compared with the WT, overexpression of *AaMYC2* significantly increased the content of artemisinin and DHAA in transgenic *A. annua*. Meanwhile, the content of artemisinic acid, which was the competitive pathway product, was significantly reduced in *AaMYC2* overexpressing lines (Shen et al., 2016).

#### Basic Leucine Zipper (bZIP) Transcription Factors

A basic leucine zipper family transcription factor (*AabZIP1*) was cloned from *A. annua*. Overexpression of *AabZIP1* upregulates the expression of *ADS* and *CYP71AV1* and promotes the biosynthesis of artemisinin in transgenic *A. annua* plants. Compared to wild-type lines, the artemisinin content increased 0.7- to 1.5-fold (Zhang et al., 2015). Hence, all these transcription factors could be plant metabolic engineering tools for sustainable production of high-value pharmaceutical terpenoids in medicinal plants.

### Regulating the Levels of Endogenous Phytohormones Involved in Terpenoid Biosynthesis

Terpenoid biosynthesis is often induced by herbivore feeding or pathogen attack (Vranová et al., 2012). The transcriptional response is controlled by a complex signaling cascade in which jasmonate hormones (JAs) play a crucial role (Moses et al., 2013; Ahmad et al., 2016). Hence, the production of pharmaceutical terpenoids may be regulated through the jasmonate biosynthetic pathway in plants. Overexpression of allene oxide cyclase gene from *S. miltiorrhiza* (*SmAOC*), the key enzyme of the jasmonate biosynthetic pathway, significantly enhanced the expression levels of genes of the diterpenoids biosynthetic pathway and increased the yields of tanshinone IIA, rosmarinic acid and lithospermic acid B in *S. miltiorrhiza* hairy root cultures (Gu et al., 2012). Jasmonates also play crucial roles in the regulation of artemisinin biosynthesis. The allene oxide cyclase gene from *A. annua* (*AaAOC*) was cloned and overexpressed in *A. annua* plants and the content of endogenous JA was increased 2- to 4.7-fold compared to the control. The increased endogenous JA promoted the expression levels of *FDS, CYP71AV1* and *DBR2*, which resulted in a significant increased production of artemisinin, DHAA and artemisinic acid (Lu et al., 2014). However, high concentrations of JA in plants may inhibit plant growth, which limit the use of this method.

The phytohormone abscisic acid (ABA), which plays an important role in plant development and environmental stress response, may also participate in the regulation of the pharmaceutical terpenoid biosynthesis. The *SlNCED1* gene, which encodes the enzyme 9-*cis*-epoxycarotenoid dioxygenase (NCED) of the ABA biosynthetic pathway, was suppressed in tomato by a fruit-specific E8 promoter driven RNAi construct. Compared to the control, *SlNCED1* transcript levels were down-regulated to 20–50% in the transgenic tomato, which partially blocked the carbon flow to free ABA and ABA metabolite accumulation. The decrease in endogenous ABA resulted in an increase in ethylene formation due to an increased transcription of ethylene biosynthesis pathway genes during ripening. The blocked carbon flow also influenced the carotenoid pathway of the RNAi lines resulting in increased lycopene and β-carotene accumulation. Therefore, tomato fruit of RNAi lines displayed deep red coloration compared to the control fruit during ripening (Sun et al., 2012). ABA treatment could also increase the production of artemisinin (Lu et al., 2011). The full-length cDNA of ABA receptor *AaPYL9* was cloned and characterized from *A. annua*. Overexpression of *AaPYL9* increases not only drought tolerance, but also artemisinin content after ABA treatment, with significant enhancement of the expression of artemisinin biosynthesis genes (Zhang et al., 2013).

### Regulating Related Primary Metabolism

Improving the productivity of pharmaceutical terpenoids can also be achieved by regulating the relevant primary metabolism of medical plants. Carbohydrates are important products of primary metabolism. A large-scale statistical experiment showed that carbohydrates played a major role in determining the geraniol yield in transgenic tobacco cell suspension cultures. Among sucrose, glucose and D-mannitol, the use of sucrose led to the highest geraniol yield and biomass accumulated in the cell cultures. Light, which can promote the synthesis of carbohydrates by photosynthesis, had also a potent effect on geraniol biosynthesis (Vasilev et al., 2014). Overexpressing the neutral/alkaline invertase gene (*NINV*), a key gene of sucrose hydrolysis, can significantly enhance the expression level of taxadiene synthase gene (*TAS*) in *T. chinensis* cells. The mean contents of seven individual taxanes including 10-deatetylbaccatin III, baccatin III, 10-deacetyl taxol, cephalomanine, 7-epi-10-deatetyl taxol, taxol, and 7-epi taxol were 2.1, 3.3, 2.2, 3.7, 3.5, 1.9 and 1.8 times higher than the controls, respectively. Thus, regulating the *TcNINV*-mediated sucrose metabolism can promote the biosynthesis of taxanes (Dong et al., 2015).

## Conclusions and Perspectives

Nowadays, more and more studies combined these metabolic regulation strategies to produce target terpenoids. For example, *Taxus* ×*media* hairy root cultures were obtained by overexpressing *TXS* gene from *Taxus baccata* but showed poor growth capacity. To resolve this problem, transgenic hairy roots were dedifferentiated to callus by hormonal treatment and cell suspension lines were obtained. After MeJA treatment, the taxane production reached the highest in the *TXS* cell line, which was 2.65-fold higher than the untransformed control (Expósito et al., 2010).

**FIGURE 3:**
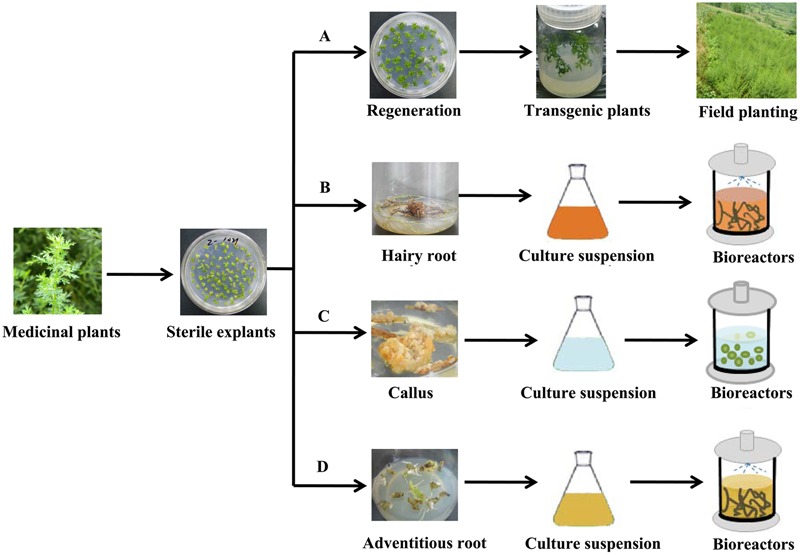
**The production of pharmaceutical terpenoids by plant metabolic engineering. (A)** The cultivation of transgenic plants **(B)** Hariy root culture **(C)** Plant cell-suspension culture **(D)** Adventitious root culture.

Plant metabolic engineering strategies hold great promise to upregulate the content of pharmaceutical terpenoids in medicinal plants. After a series of metabolic regulations in medicinal plants, the production of pharmaceutical terpenoids can be greatly improved. Compared with the control, transgenic plants, which integrate aimed DNA into the host genome by the tumor inducing (Ti) plasmid, had significantly higher pharmaceutical terpenoids. And those transgenic plants can greatly improved the production of pharmaceutical terpenoids (**Figure 3A**). Compared to cultivation of plants, plant cell cultures, including hariy root cultures, cell-suspension cultures and adventitious root cultures, have some advantages to produce pharmaceutical terpenoids (**Figures 3B–D**). The most important advantage is that the target terpenoids can be harvested under controlled conditions (Bioreactor or Flask) and with strict quality control. The growth cycles are much faster than cultivation of the plant, and can be measured in weeks rather than years (Rao and Ravishankar, 2002). However, in some cases plant cell cultures do not produce the target compounds, which limit the applicability of this approach.

Many of these pharmaceutical terpenoids are complicated compounds, and the biosynthetic pathways are not resolved. The focus of plant metabolic engineering to produce pharmaceutical terpenoids has shifted to reveal different TPSs, CYP450s, glucosyltransferases and dehydrogenases involved in the biosynthesis pathways of pharmaceutical terpenoids and to explore overexpression of transcription factor involved in the regulation of terpenoid biosynthesis. With the continuous development of plant metabolic engineering, more and more high value pharmaceutical terpenoids will be upregulated in the future. We believe that all those strategies will enhance the supply of scarce drugs, reduce the price of expensive drugs and improve people’s standards of living.

## Author Contributions

XL wrote the manuscript; KT and PL conceived the idea of this review, developed the ideas, designed the overall concept and revised the manuscript.

## Conflict of Interest Statement

The authors declare that the research was conducted in the absence of any commercial or financial relationships that could be construed as a potential conflict of interest.
